# Integrative In Vivo Drug Testing Using Gene Expression Signature and Patient-Derived Xenografts from Treatment-Refractory HER2 Positive and Triple-Negative Subtypes of Breast Cancer

**DOI:** 10.3390/cancers11040574

**Published:** 2019-04-23

**Authors:** Jin-Sun Ryu, Sung Hoon Sim, In Hae Park, Eun Gyeong Lee, Eun Sook Lee, Yun-Hee Kim, Youngmee Kwon, Sun-Young Kong, Keun Seok Lee

**Affiliations:** 1 Center for Breast cancer, National Cancer Center, Goyang 10408, Korea; 71360@ncc.re.kr (J.-S.R.); simsh@ncc.re.kr (S.H.S.); parkih@ncc.re.kr (I.H.P.); 12772@ncc.re.kr (E.G.L.); eslee@ncc.re.kr (E.S.L.); ymk@ncc.re.kr (Y.K.); 2 Division of Translational Science, National Cancer Center, Goyang 10408, Korea; 3 Department of Cancer Biomedical Science, Graduate School of Cancer Science and Policy, Goyang 10408, Korea; sensia37@ncc.re.kr; 4 Division of Convergence Technology, National Cancer Center, Goyang 10408, Korea; 5 Department of Laboratory Medicine, Center for Diagnostic Oncology, National Cancer Center, Goyang 10408, Korea

**Keywords:** patient-derived xenograft, breast cancer, pre-clinical drug test, personalized therapy, sorafenib, everolimus, docetaxel, bevacizumab, PX-478, neratinib

## Abstract

Patient-derived xenografts (PDXs) are powerful tools for translational cancer research. Here, we established PDX models from different molecular subtypes of breast cancer for in vivo drug tests and compared the histopathologic features of PDX model tumors with those of patient tumors. Predictive biomarkers were identified by gene expression analysis of PDX samples using Nanostring nCount cancer panels. Validation of predictive biomarkers for treatment response was conducted in established PDX models by in vivo drug testing. Twenty breast cancer PDX models were generated from different molecular subtypes (overall success rate, 17.5%; 3.6% for HR^+^/HER2^−^, 21.4% for HR^+^/HER2^+^, 21.9% for HR^−^/HER2^+^ and 22.5% for triple-negative breast cancer (TNBC)). The histopathologic features of original tumors were retained in the PDX models. We detected upregulated HIF1A, RAF1, AKT2 and VEGFA in TNBC cases and demonstrated the efficacy of combined treatment with sorafenib and everolimus or docetaxel and bevacizumab in each TNBC model. Additionally, we identified upregulated HIF1A in two cases of trastuzumab-exposed HR^−^/HER2^+^ PDX models and validated the efficacy of the HIF1A inhibitor, PX-478, alone or in combination with neratinib. Our results demonstrate that PDX models can be used as effective tools for predicting therapeutic markers and evaluating personalized treatment strategies in breast cancer patients with resistance to standard chemotherapy regimens.

## 1. Introduction

Developing models that mimic human cancer is a long-standing goal in cancer research [1]. By testing treatments in a model similar to a given patient’s condition, it should be possible to identify personalized, effective treatment options that avoid unnecessary therapeutic interventions.

Cancer cell lines are among the most popular tools for drug testing and molecular research because of their ease of handling and rapid proliferation. However, such cell lines are in vitro models that have a limited ability to recapitulate the features of actual cancers. A xenograft model generated by injection of a cancer cell line into immunodeficient mice is a widely used model for preclinical drug studies [2]. However, xenograft models created using cancer cell lines do not reflect the heterogeneity of complex tumors and often do not reproduce the microenvironment of tumor cells in vivo, including accessory cell components such as fibroblasts, blood vessel cells and immune cells [3,4].

To overcome these limitations, researchers have increasingly turned to patient-derived xenograft (PDX) models as a useful platform for translational and preclinical research in general oncology, including breast cancer. PDX models are known to exhibit histologic characteristics and mutational profiles similar to those of patient cancers from which they were derived [5,6,7,8,9,10,11]. Such models can also amplify the amount of tumor tissue available and create an in vivo drug testing environment for further research. Technological advances have created the three-dimensional (3D) culture systems, patient-derived organoids that better mimic the physiological state of organs and tissues [12]. They are relatively easy to generate, rather than using animal models for cancer research. However, organoids lack the stromal components, vessels and other microenvironments surrounding tumors [13].

Various cancer drugs have been developed for treating breast cancer and numerous clinical trials for new agents are ongoing. In the clinic, it is important to select the anticancer agents most effective for an individual patient. Thus, a model capable of predicting the effectiveness of anticancer agents would be invaluable in drug development and patient treatment. It is relatively easy to obtain tumor tissue from breast cancer for the establishment of PDX models because the clinical course of the disease has a long trajectory from diagnosis to death. Considering the time required for PDX establishment, breast cancer is a good cancer type for clinical application of PDX research.

Here, we developed PDX models from different molecular subtypes of breast cancer. This study showed the feasibility of drug testing and confirmed the potential activity of combination treatments in PDX models from different breast cancer subtypes for personalized therapy.

## 2. Results

### 2.1. Patient Characteristics and PDX Establishment

**Figure 1:**
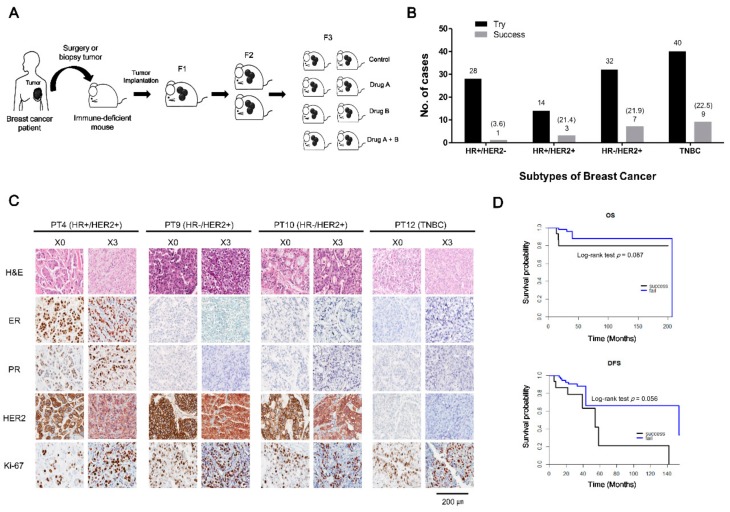
Establishment of patient-derived xenograft (PDX) models from different molecular subtypes of breast cancer. (**A**) Scheme of breast cancer PDX establishment and drug treatment. Surgical or biopsy tumor samples from breast cancer patients were implanted into the mammary fat pads of immunodeficient mice. Following growth of the tumor in passage 1 (F1), tumor fragments were serially transferred from mouse to mouse. In drug-response tests, PDXs were treated orally or intraperitoneally with drug A, B, or their combination. Drug efficacy was assessed by monitoring tumor growth, validating histological features, and determining expression of target proteins. (**B**) PDX engraftment patterns according to breast cancer subtype. The number of PDX implantation trials and successes according to breast cancer subtype in samples obtained at the National Cancer Center, Korea, between July 2014 and September 2017. Success rates of PDX engraftment according to molecular subtype are shown in parentheses. The PDX engraftment rate for HR^−^/HER2^+^, HR^+^/HER2^+^ and TNBC subtypes was relatively high (~21%) compared with that of the HR^+^/HER2^−^ subtype. (**C**) Comparison of histological features between representative patients’ original tumors and the corresponding PDX tumors. Four representative patients’ xenograft tumors showing retention of the same morphological features (H&E staining) and ER, PR, HER2 and Ki-67 biomarker status as the original tumors. Patient samples are shown in the left column; corresponding xenograft results are depicted in the right column. Scale bar, 200 μm. (**D**) Overall survival (OS) and disease-free survival (DFS) of breast cancer patients according to PDX engraftment status. OS and DFS were analyzed using Kaplan–Meier plots and the log-rank test. Tumors from patients with poor OS (*p* = 0.087) and DFS (*p* = 0.056) showed a good engraftment tendency.

Tumor samples were obtained from 114 breast cancer patients at National Cancer Center, Korea, and were implanted into the mammary fat pads of immunodeficient mice (Figure 1A). Of the 114 implanted samples, 20 successfully established PDX models (overall engraftment rate, 17.5%). The success rates according to breast cancer subtype were 3.6% for HR^+^/HER2^−^, 21.4% for HR^+^/HER2^+^, 21.9% for HR^−^/HER2^+^, and 22.5% for triple-negative breast cancer (TNBC) (Figure 1B). Clinical features of patients according to PDX engraftment status are shown in Table 1 and Table S1. Patient age and source of engraft tissue which came from surgical specimens or biopsy samples did not affect PDX engraftment (*p* = 0.175, and *p* = 0.085, respectively). The stage distribution was significantly different between the two groups (*p* = 0.039), with a greater representation of advanced stages in the successfully engrafted group. The success rate showed a tendency to increase according to T stage of patients as follows: 7.1% in T1, 11.7% in T2, 23.1% in T3, and 33.3% in T4. However, it was not statistically significant (*p* = 0.185). Chemotherapy resistance, defined as cancer progression during chemotherapy or recurrence within one year after adjuvant chemotherapy, was also significantly associated with successful engraftment (*p* < 0.001). An analysis of PDX engraftment according to survival showed that tumors derived from patients with poor disease-free and overall survival showed a tendency towards good engraftment (Figure 1D). The time required to reach 100 mm^3^ in the first generation (F1) after implantation averaged 62 days, and there was no difference in the rate of stable transplantation by molecular subtype (*p* = 0.830) (Table S1).

### 2.2. Histologic Characteristics of Patient Breast Cancers and Their Corresponding Xenografts

To confirm that histological features of the original tumor were stably retained in the xenograft, we conducted a comparative histological analysis of xenograft tumors and tumors of origin using ER, PR, HER2 and Ki67 as biomarkers. Figure 1C shows histological results of representative patients and their xenograft biopsies. For each subtype, morphological characteristics were conserved and hormone receptor, HER2 and Ki-67 statuses were maintained throughout xenograft passages. These data confirm that the histological features of the original tumors were conserved in xenografts and that the PDX models of breast cancer developed in this study reflect the characteristics of the original tumors.

### 2.3. Drugs Response in PDX Models: TNBC Cases

**Figure 2:**
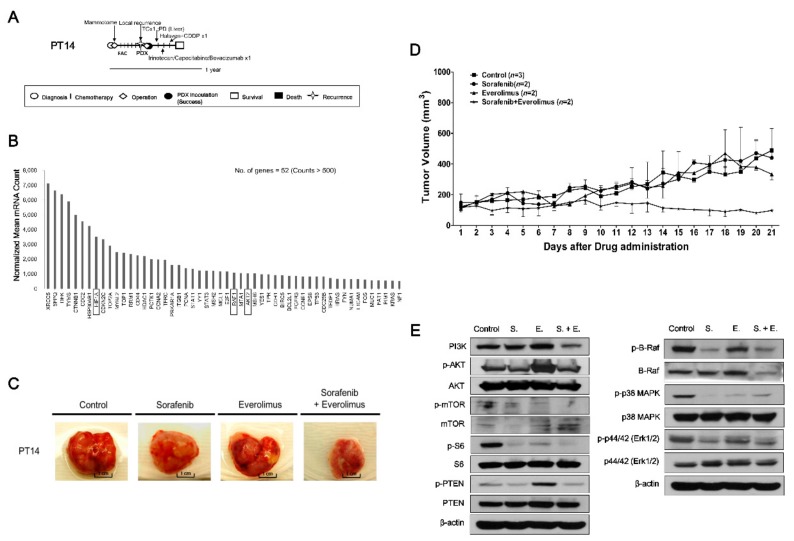
In vivo efficacy of sorafenib and everolimus against PDX models from PT14 (TNBC subtype). (**A**) PT14 treatment history. See text for details. Scale bar, one year. (**B**) Gene expression analysis using Nanostring nCounter GX human cancer reference kit in the PDX model (F1) from PT14. Expression data were analyzed using the nSolver analysis module. Expression values (nCounts) were normalized to six internal reference genes. The top upregulated 52 genes with counts >500 were selected among the 230 human cancer-related genes. (**C**) Images of PDX model tumors from PT14 following treatment with sorafenib and everolimus, alone and in combination. (**D**) Tumor volumes (F3) were determined in female mice (*n* = 2–3) treated with sorafenib (●, 120 mg/kg), everolimus (▲, 20 mg/kg), or their combination (★), administered once daily for three weeks by oral gavage; mice administered saline (■) served as controls. Tumor volumes at 21 days are presented as means ± SD (*p* < 0.05 for control vs. sorafenib + everolimus; unpaired *t*-test). (**E**) Expression of PI3K, p-AKT, AKT, p-mTOR, mTOR, p-S6, S6, p-PTEN, PTEN, p-B-Raf, B-Raf, p-p38 MAPK, p38 MAPK, p-p44/42, p44/42, and β-actin proteins in PDX tumors from PT14 treated with sorafenib, everolimus, and their combination. S.; sorafenib, E.; everolimus, S. + E.; sorafenib + everolimus.

Patient 14 (PT14, F/27), diagnosed with TNBC (pT1cN0), received breast-conserving surgery followed by adjuvant fluorouracil (FAC) chemotherapy. One month later, local recurrence was detected (rpT3N2) and a modified-radical mastectomy (MRM) was performed, at which point tumor tissue was taken for PDX establishment. Despite administration of multiple chemotherapies, including docetaxel, capecitabine/irinotecan with bevacizumab, and eribulin with cisplatin, the disease spread throughout the body within six months (Figure 2A). To explore novel therapeutic targets in TNBC tumors, we analyzed gene expression patterns in PDX model established from the TNBC tumor of PT14 using a Nanostring nCounter GX Human Cancer Reference kit consisting of 230 human cancer-related genes and six internal reference genes (nCounts > 500) (Figure 2B). Based on gene expression and drug availability, sorafenib and everolimus were selected for targeting activated hypoxia inducible factor 1 subunit alpha (HIF1A), the proto-oncogene RAF1, and the serine/threonine kinase AKT2 in the PDX tumors developed from PT14 (Figure 2). After three weeks per oral administration, the tumor volume was reduced only by combined treatment with sorafenib and everolimus (control vs. sorafenib + everolimus, *p* = 0.042), which was tolerated since there was less than a 15% loss of body weight (Figure 2C,D and Figure S1A). The expression levels of PI3K/AKT/mTOR and MAPK signaling proteins were reduced in the group treated with the combination of sorafenib and everolimus compared with the control group (Figure 2E).

**Figure 3:**
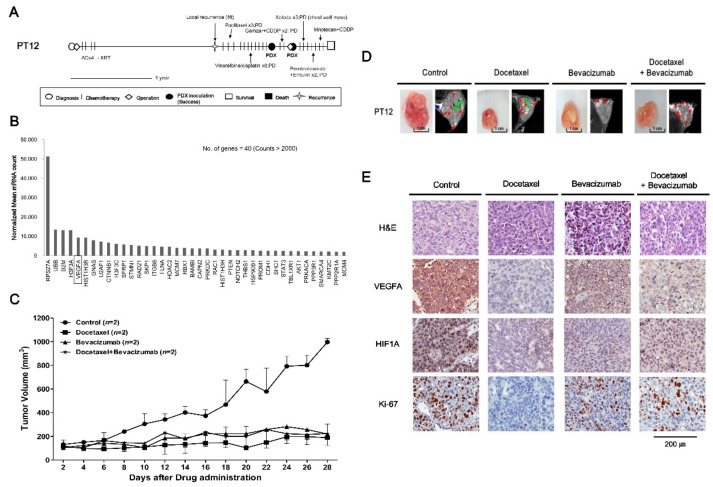
In vivo efficacy of docetaxel and bevacizumab against PDX models from PT12 (TNBC subtype). (**A**) PT12 treatment history. See text for details. Scale bar, one year. (**B**) Gene expression analysis (F3) using a NanoString PanCancer Pathway panel. The top upregulated 40 genes with counts >2000 were selected among the 730 human cancer-related genes. (**C**) Tumor volumes (F3) were determined in female mice (*n* = 2) intraperitoneally injected with docetaxel (3 mg/kg), bevacizumab (5 mg/kg), or their combination three times a week for 4 weeks; mice injected with saline served as controls. Tumor volumes at 28 days are presented as means ± SD (*p* ≤ 0.05 for control vs. docetaxel and control vs. bevacizumab; *p* = 0.06 for control vs. docetaxel + bevacizumab; unpaired *t*-test). (**D**) MRI images of tumors 28 days after drug administration. Red arrows indicate necrosis, green arrows are traces of blood flow, and blue arrows represent fat. (**E**) Histology and immunohistochemistry of PT12 PDX models. H&E staining and expression of Ki-67 (cell proliferation marker), VEGFA (the marker of angiogenesis), and HIF1A (upstream regulator of VEGFA) in PDX tumors treated with individual drugs or drug combinations were compared with controls. Scale bar, 200 μm.

PT12 (F/35), diagnosed with TNBC (pT2N0), received breast-conserving surgery, adjuvant chemotherapy, and radiation therapy. One year later, local recurrence was detected. Although multiple chemotherapies were administered, including paclitaxel, vinorelbine with cisplatin, gemcitabine with cisplatin, capecitabine and eribulin, the disease progressed and metastasized (Figure 3A). To further test the antitumor effects of combined treatment with sorafenib and everolimus in the TNBC subtype, we treated the PT12 PDX model with sorafenib and everolimus, alone and in combination (Figure S2). Each treatment regimen tended to diminish the growth of PDX tumors derived from PT12; although the trend was stronger for single treatment, none of these treatments caused a significant effect (*p* > 0.2 for control vs. sorafenib, everolimus and sorafenib + everolimus) (Figure S2A,B). Treatment with sorafenib or the combination of sorafenib with everolimus resulted in no significant differences in the detected expression levels of Ki-67, VEGFA, or HIF1A between control and drug-treated tumors (Figure S2C). To explore potentially more effective treatments specific to PT12, we analyzed gene expression patterns for PT12 PDX tumors using the Nanostring PanCancer Pathway panel (Figure 3B). This analysis revealed that VEGFA was among the most highly expressed genes (nCounts > 2000) (Figure 3B). On the basis of previous studies that have targeted VEGFA in breast cancer [14,15,16,17], we tested the tumor-suppressive effects of bevacizumab, a humanized Vascular Endothelial Growth Factor A (VEGFA) antagonist and docetaxel, which was chosen with the consideration of treatment history on PDX models derived from PT12 (Figure 3). The combination of bevacizumab and docetaxel were widely used in metastatic breast cancer treatment [18]. Treatment with docetaxel or bevacizumab individually for 28 days significantly decreased volumes of PDX model tumors from PT12; drug treatment was well tolerated as indicated by the lack of significant body weight loss (*p* ≤ 0.05 for control vs. docetaxel and control vs. bevacizumab) (Figure 3C,D and Figure S1B). However, combination therapy with bevacizumab and docetaxel did not exert synergistic tumor growth inhibition effects compared with single drug treatment (control vs. docetaxel + bevacizumab, *p* = 0.06). The number of proliferating (i.e., Ki-67-expressing) cells was reduced in bevacizumab, docetaxel, and the combination treatment groups compared with the control group (Figure 3E). Treatment with bevacizumab, docetaxel, or their combination also suppressed the expression of VEGFA and HIF1A, factors upstream of VEGFA in hypoxia signaling, particularly treatment with docetaxel (Figure 3E). These results support the conclusion that targeting of VEGF would be an effective therapeutic strategy for TNBC patient PT12.

### 2.4. Evaluating Anticancer Drug Effects in Trastuzumab-Exposed PDX Models: Cases of HR^−^/HER2^+^ Breast Cancer

**Figure 4:**
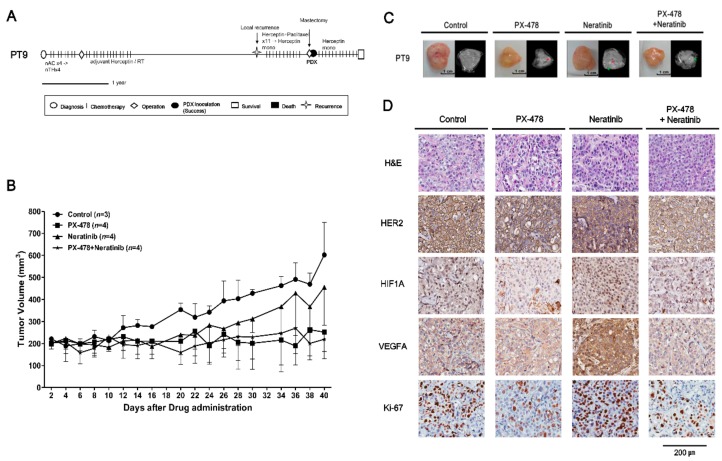
In vivo efficacy of PX-478 and neratinib against PDX models from PT9 (HR^−^/HER2^+^ subtype). (**A**) PT9 treatment history. See text for details. Scale bar, one year. (**B**) Tumor volumes (F3) were determined in female mice (*n* = 3–4) administered PX-478 (10 mg/kg), neratinib (20 mg/kg), or their combination three times a week for 40 days by gavage; mice administered saline served as controls. Tumor volumes at 40 days are presented as means ± SD (*p* ≤ 0.05 for control vs. PX-478 and control vs. PX-478 + neratinib; *p* = 0.28 for control vs. neratinib; unpaired *t*-test). (**C**) MRI images of tumors 30 days after administration of drugs. Red arrows indicate necrosis and green arrows are traces of blood flow. (**D**) H&E staining and immunohistochemical analyses of HER2, Ki-67, VEGFA and HIF1A in PDX tumors from PT9 after treatment with drugs. Scale bar, 200 μm.

**Figure 5:**
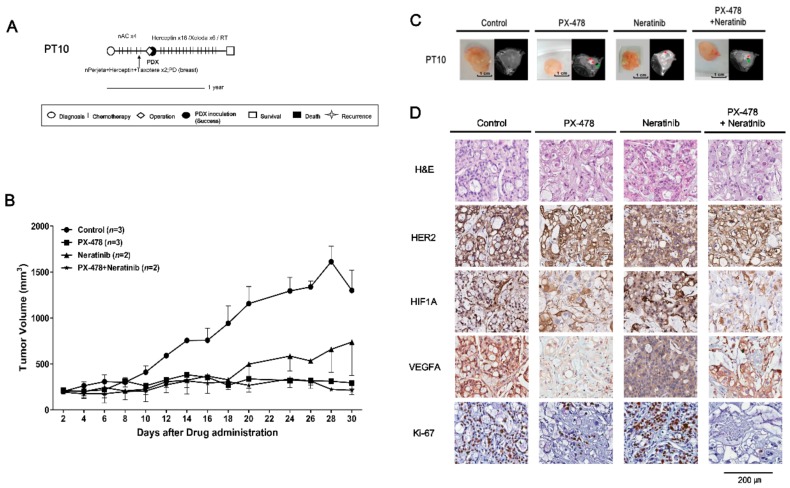
In vivo efficacy of PX-478 and neratinib against PDX models from PT10 (HR^−^/HER2^+^ subtype). (**A**) PT10 treatment history. See text for details. Scale bar, one year. (**B**) Tumor volumes (F3) were determined in female mice (*n* = 2–3) given PX-478 (10 mg/kg), neratinib (20 mg/kg), or their combination three times a week for 30 days by peroral administration; mice administered saline served as controls. Tumor volumes at 30 days are presented as means ± SD (*p* ≤ 0.05 for control vs. PX-478 and control vs. PX-478 + neratinib; *p* = 0.32 for control vs. neratinib; unpaired *t*-test). (**C**) MRI images of tumors at 30 days after administration of drugs. Red arrows represent necrosis and green arrows are traces of blood flow. (**D**) H&E staining and immunohistochemical analyses of HER2, Ki-67, VEGFA and HIF1A in PDX tumors from PT10 after treatment with drugs. Scale bar, 200 μm.

PT9 (F/39) received neoadjuvant chemotherapy, modified radical mastectomy (MRM), radiation therapy and adjuvant trastuzumab. Several years later, local recurrence was detected. After paclitaxel with trastuzumab was administered, palliative mastectomy was performed with concurrent tissue sampling for PDX establishment. PT9 has continued to receive palliative treatment (Figure 4A). PT10 (F/46) received neoadjuvant chemotherapy with docetaxel, trastuzumab and pertuzumab. Following treatment, the disease progressed and an MRM was performed with concurrent tissue sampling for PDX establishment. PT10 is currently in follow-up without disease recurrence (Figure 5A).

To optimize the therapeutic strategies for trastuzumab-exposed HR^−^/HER2^+^ breast cancer patients, we established PDX models from HR^−^/HER2^+^ tumors from PT9 and PT10. We performed a multiplex gene expression analysis using the Nanostring nCounter GX Human Cancer Reference kit for PT5 with a HR^−^/HER2^+^ subtype, which revealed high expression of HIF1A and HER2/ERBB2 (nCounts >500) (Figure S3A). Initially, the PDX model from PT5 was used to detect the potential drug targets, and test the tumor growth inhibitory effects of PX-478 (S-2-amino-3-(4′-N,N,-bis(chloroethyl)amino) phenyl propionic acid N-oxide dihydrochloride), a small molecule that selectively inhibits hypoxia-induced HIF1A mRNA expression and translation [19,20,21], and neratinib on this HR^-^/HER2^+^ subtype. PX-478 and the combination of PX-478 with neratinib significantly inhibited tumor growth compared with control and neratinib treatment groups (Figure S3B). Immunohistochemical data showed that the expression level of HIF1A was also highly increased in PT9 and PT10 PDX models (Figure 4 and Figure 5D). On the basis of these results, we tested PX-478 (10 mg/kg), neratinib (20 mg/kg), and their combination in PT9 and PT10 PDX models (Figure 4 and Figure 5). Notably, PX-478 alone and the combination of PX-478 with neratinib showed significant tumor growth inhibition (*p* < 0.05) in both two trastuzumab-exposed HR^−^/HER2^+^ PDX models compared with controls (Figure 4 and Figure 5B,C). However, treatment with neratinib alone did not cause significant tumor growth inhibition (*p* = 0.28). Treatment of PDX tumor-bearing mice with these drugs caused less than a 15% reduction in body weight (Figure S1C,D), suggesting minimal toxicity. An immunohistochemical analysis suggested that PX-478 alone or in combination with neratinib more reduced Ki-67 expression than that of control or neratinib treated groups (Figure 4 and Figure 5D). HER2 expression level was not different between neratinib treatment groups and other drug treatment groups (Figure 4 and Figure 5D). HIF1A expression was attenuated by PX-478 alone and combined treatment with PX-478 and neratinib, whereas it was unaffected by treatment with neratinib alone (Figure 4 and Figure 5D). VEGFA expression, a downstream target of HIF1A, was only reduced by treatment with PX-478 alone and combination treatment groups in PT10 PDX model (Figure 4 and Figure 5D). These data suggest that inhibition of HIF1A is an alternative therapeutic strategy for trastuzumab-exposed HR^−^/HER2^+^ breast cancer tumors.

## 3. Discussion

In this study, we successfully established PDX models from various subtypes of breast cancer and demonstrated that PDX model tissues bore the same histological characteristics as the primary tumors from which they were derived. On the basis of an analysis of tumor gene expression, we selected potential treatment targets and drugs. Using these PDX models, it was possible to perform successful in vivo drug tests on HR^−^/HER2^+^ and TNBC subtypes.

PDX models had been known to be one of the good tools mimicking biological characteristics of cancers [22]. These sustain histological characteristics and functions after mice passages. However, it is relatively difficult to engraft the tumors in the immunodeficient mice and it takes time to develop a successful PDX model, which usually takes 4–8 months to go through a few generations [23]. Those are big obstacles in developing a personalized treatment strategy for each patient. PDX engraftment success rates in previous studies have been shown to vary, ranging from approximately 15% to 30% [10,23,24]. The tumor tissue factors that affect successful engraftment are still not well known. However, it has been shown that aggressive tumor characteristics are associated with engraftment success. One of the factors indicative of tumor aggressiveness is breast cancer tissue subtype. Previous reports have shown relatively high success rates for HER2-positive and TNBC types, and low success rates for the HR-positive subtype [10,23,24]. In the present study, the success rate of PDX establishment according to subtype was 21.9% for HR^−^/HER2^+^, 22.5% for TNBC, and 3.6% for HR^+^/HER2^−^, results compatible with previous reports [10,23,24,25,26]. Another aggressive tumor feature is chemotherapy resistance. In the samples derived from chemotherapy-resistant tumors, the PDX success rate was relatively high compared with that in samples lacking chemotherapy resistance, indicating that resistance to treatment can also affect PDX success rate. Other factors affecting success rate may be tumor microenvironment and mouse strain. Co-introduction of an immortalized human fibroblast and mesenchymal stem cell were explored to improve PDX success rate [27,28]. Various mouse strains with different immunosuppression can also affect the outcome [29]. No specific methods have been established, thus further studies are required for enhancing the clinical utility of PDX models.

To investigate new therapeutic strategies for treatment-resistant breast cancer, we explored treatment targets using multigene panels, which showed high expression of HIF1A, RAF1, AKT2 and VEGFA in two TNBC cases. Among those genes, targets and drugs were chosen on the basis of the gene expression level with related pathways, drug availability in clinics and in vivo models and treatment history. As treatment options, we tested two drugs. The first, sorafenib, is a small molecule inhibitor of Raf kinase, VEGF receptor (VEGFR)-2 and -3 and platelet-derived growth factor (PDGF) tyrosine kinases that target tumor cell proliferation and angiogenesis [30,31]. The second, everolimus, inhibits mTOR kinases that act in PI3K/AKT/mTOR signaling pathways, which are important for cell growth and survival [32]. mTOR inhibitors also inhibit HIF1A translation [33,34]. Treatment of the PDX from PT14 with both drugs inhibited PI3K/AKT/mTOR and MAPK signaling pathways and decreased PDX tumor volume, suggesting a potential new treatment option for TNBC.

Increased VEGFA was identified as a good treatment target in PT12 (TNBC subtype) by a Nanostring Pancancer panel analysis. VEGFA, which is a major factor in angiogenesis, binds to and acts on both VEGFR-1 and VEGFR-2 to stimulate angiogenesis, vascular permeability and gene expression [14,15]. Treatment with sorafenib and everolimus inhibited the growth of the PDX model tumors from PT12, but treatment with sorafenib, an inhibitor of VEGFR2 and -3 tyrosine kinase activity, did not decrease expression of VEGFA in tumors (Figure S2C). This could be a reflection of the limited effectiveness of sorafenib against VEGFA in PT12. Previous studies have reported that progression-free survival in advanced HER2-negative breast cancer is improved by combined treatment with docetaxel and bevacizumab [17,35], the latter of which is a well-known antibody that inhibits interactions between VEGF ligands and their receptors [16]. Treatment with bevacizumab or docetaxel reduced the volume of PDX tumors from PT12, but the combination of both drugs did not exert synergistic tumor growth inhibition. An IHC analysis of markers revealed that Ki-67, VEGFA and HIF1A expression were decreased in the corresponding tumors in tumor-bearing mice treated with bevacizumab, docetaxel, or their combination (Figure 3). These results suggest that direct targeting of VEGFA using bevacizumab in PDX tumors from PT12 is a better treatment strategy than sorafenib-mediated inhibition of VEGFR-2 and-3 tyrosine kinases.

In HR^−^/HER2^+^ cases, HIF1A was selected as a treatment target based on gene expression analyses. Previous studies have reported that HIF1A levels are increased in SKBR3 cells (HR^−^/HER2^+^), and that constitutively active HER2 is expressed in MCF-10A cells under normoxic conditions [36]. HIF1A protein is also expressed at high levels in SKBR3 compared with the other breast cancer cell lines, MDA-MB-231 (TNBC) and T47D (HR^+^/HER2^−^), under normal culture conditions [37]. We also confirmed that HIF1A expression level was elevated in PDX tumors from PT9 and PT10 (HR^−^/HER2^+^) (Figure 4 and Figure 5D). IHC results obtained in PT9 and PT10 PDX models showed that tumor growth and the expression of HIF1A, VEGFA1 and Ki-67 were efficiently suppressed by treatment with PX-478, alone or in combination with neratinib (Figure 4 and Figure 5). These results imply that HIF1A may be a novel, alternative therapeutic target in trastuzumab-exposed HR^−^/HER2^+^ breast cancer.

In this study, we identified gene expression related to treatment targets in each PDX model. PDX models derived from different patients with the same subtypes did not always show consistent responses following treatment with the same drugs. This variability may be attributable to the selection of drugs based solely on gene expression patterns in PDX tumors from patients. The analysis of gene expression indirectly may reflect the genetic variations of the tumors, since the variants affect the gene regulatory functions [38]. However, a more thorough analysis of genetic alterations could improve the selection of effective drugs for each patient.

Although we explored appropriate treatments for individual patients using PDX models, in five cases we failed to reach the clinical application stage. Moreover, the majority of patients with successfully established PDX models died or showed poor prognosis and could no longer use the new treatments. Considering the short survival time of metastatic cancer patients, the time required to establish PDX is a hurdle for the application of PDX in individual patient treatment decisions. Another difficulty is the use of off-label cancer drugs without a clinical trial program. Despite these limitations in directly applying results to the clinic, the PDX model can be a good tool for exploring new treatments for patients who show resistance to standard therapies.

## 4. Materials and Methods

### 4.1. Patient Samples

Surgery or biopsy tumor samples were collected from patients with stage 1–4 breast cancer treated at the National Cancer Center, Korea, between July 2014 and September 2017. Tumor samples were tested for estrogen receptor (ER) status (+/−), progesterone receptor (PR) status (+/−), and HER2 (human epidermal growth factor receptor 2, also known as ERBB2) status (+/−) using immunohistochemistry (IHC) and fluorescence in situ hybridization (FISH) approaches [39,40]. Receptor positivity was accessed according to the 2010 and 2013 American Society of Clinical Oncology (ASCO) College of American Pathologists (CAP) guidelines [41,42]. Histopathological morphology of xenografts was also reviewed along with receptor status and was compared with that of patient tumors. Proliferation was assessed based on the area with the most intense staining for the proliferation marker, Ki-67. Breast cancer patient tumors were classified into four groups according to the IHC and FISH results: HR^+^/HER2^−^, HR^−^/HER2^+^, HR^+^/HER2^+^ and triple-negative breast cancer (TNBC). This study was approved by the Institutional Review Board of the National Cancer Center, Korea (NCC2014-0125, NCC2016-0091, NCC2017-0146), and written consent was obtained from all patients.

### 4.2. Generation of PDX Breast Cancer Models

The experimental protocol for this study was approved by the Institutional Animal Care and Use Committee (No. NCC-14-224, NCC-16-224, NCC-17-224). Surgically resected tumors or tumor biopsies were implanted into the mammary fat pad of 6-week-old female athymic Nude-Foxn1nu (Envigo, Tokyo, Japan), NOD/SCID/IL2Rγ-null, NSG or NOG mice (CIEA, Kawasaki, Japan) under isoflurane anesthesia (Hana Pharm Co. Ltd., Hwasung, Korea). Seven days before tumor inoculation, a 17β-estradiol pellet (Innovative Research of America, Sarasota, FL, USA) was subcutaneously implanted into the dorsal flank to support establishment of ER-positive tumors. These established PDX models were defined as passage 1 (F1). After inoculated tumors reached approximately 100–200 mm^3^ in size, they were collected, cut into ~20 mm^3^ fragments, and transferred to a sterile petri dish containing phosphate-buffered saline (PBS). Fragments of 40–60 mm^3^ were then transplanted into new recipient mice, a process that was serially repeated. The established PDX models were maintained at the National Cancer Center animal facility (Goyang, Korea).

### 4.3. In Vivo Drug Treatment

The anti-cancer agents for drug testing were selected according to the following: (1) Gene expression that can be potentially targeted or that can be related to specific drug activity and (2) the presence of available drugs and clinical decision based on treatment history. The following drugs were used for in vivo treatment of tumor-bearing mice (low passage PDXs, ≤F4): The HIF1-inhibitor, PX-478 (10 mg/kg, by oral gavage, three times a week for 2 months; Selleckchem, Houston, TX, USA); the EGFR/ERBB2 inhibitor, neratinib (20 mg/kg, orally, three times per week for 2 months; Selleckchem, Houston, TX, USA); docetaxel (3 mg/kg, by intraperitoneal administration, twice weekly for 4 weeks; Selleckchem, Houston, TX, USA); the anti-VEGF (vascular endothelial growth factor) monoclonal antibody, bevacizumab (5 mg/kg, by intraperitoneal administration, twice weekly for 4 weeks; Roche, Basel, Switzerland); the VEGFR/PDGFR/Raf inhibitor, sorafenib (120 mg/kg, via oral gavage, daily for 3 weeks; Bayer, Leverkusen, Germany); and the mTOR inhibitor, everolimus (20 mg/kg, by oral gavage, daily for 3 weeks; Novartis, Basel, Switzerland). No specific strategy for randomization was applied, and no sample size calculations were performed. The tumor sizes of mice (*n* = 2–4/group) were measured three times a week and mean volumes were plotted. After completing drug treatment of PDX tumors, mice were examined using a 7T Biospec 70/20 USR MR imaging system (Bruker, Billerica, MA, USA). Animals were monitored daily for adverse effects from tumor growth or treatment based on normal behavior, including mobility, food and water intake, weight gain/loss, and other abnormal effects. Death and clinical manifestations were all recorded.

### 4.4. Histologic Evaluation of PDX Tumors

Tumor morphology was assessed by hematoxylin and eosin (H&E) staining, and expression of the biomarkers, ER, PR, HER2 and Ki-67, was evaluated by IHC. Briefly, tumors and organs were fixed in a 10% formalin solution for 48 h. After fixation, tumors and organs were embedded in paraffin and maintained at room temperature. Sections (4 μm thick) were cut from paraffin blocks using an HM 340E Electronic Rotary Microtome (Thermo-Fisher, Walldorf, Germany) and mounted on silane-coated glass slides. Sections were incubated at 60 °C, deparaffinized in xylene, and rehydrated in a graded ethanol series. For antigen retrieval, slides were placed in boiled 10 mM citrate buffer for 20 min, then washed with running tap water for 5 min and 1× PBS for 5 min. Sections were blocked by incubating with a peroxidase-blocking reagent for 15 min at room temperature, washed for 10 min, then treated with protein blocking solution for 10 min to prevent non-specific protein binding. After blocking, sections were incubated at 4 °C overnight with primary antibodies against ER, PR, HER2, Ki-67 (Ventana Medical Systems, Tucson, AZ, USA), VEGFA (Santa Cruz biotechnology, Dallas, TX, USA), and HIF-1A (Abcam, Bristol, UK). Immunoreactive proteins were then detected using an Expose Mouse and Rabbit Specific HRP/DAB Detection IHC Kit (Abcam). Images of slide-mounted sections were acquired with a digital slide scanner (ScanScope XT; Aperio Technologies Inc., Vista, CA, USA). Biomarker expression patterns in PDX tumors were compared with the clinical pathology results of the origin tumors.

### 4.5. Detection of Target Genes in PDX Tumors Using Nanostring multigene panels

Frozen primary tumors from patients and derived xenografts were ground in liquid nitrogen, and RNA/DNA was isolated from the resulting powder using an Allprep DNA/RNA Mini kit (Qiagen Inc., Germantown, MD, USA) according to the manufacturer’s instructions. RNA quality was examined using an Agilent 2100 Bioanalyzer (RNA 6000 Nano chip; Agilent Technologies, Santa Clara, CA, USA). Gene expression signatures of PDX tumors were evaluated using an nCount GX Human Cancer Reference Kit for PT5, PT14 and nCount NanoString PanCancer Pathway panel for PT12 (NanoString Technologies Inc., Seattle, WA, USA). Approximately 100–300 ng of total RNA was processed according to the manufacturer’s protocol and gene expression was assessed using a Nanostring nCounter. Raw data (nCount) were generated with nSolver 4.0 (NanoString Technologies Inc., Seattle, WA, USA) and normalized against 40 reference genes.

### 4.6. Evaluation of Target Protein Responses to Drug Treatment by Western Blotting

The protein concentration in tissue lysates was quantified using a bicinchoninic acid assay (BCA) Protein Assay kit (Thermo Scientific, Rockford, IL, USA). 50 μg proteins were separated by sodium dodecyl sulfate-polyacrylamide gel electrophoresis (SDS-PAGE) using 8–12% polyacrylamide gels, and then transferred to polyvinylidene difluoride membranes. After blocking in 5% milk for 2 h at room temperature, the membranes were incubated overnight at 4 °C with primary antibodies (diluted 1:1000) against phosphoinositide 3-kinase (PI3K), AKT, p-AKT (Ser473), mammalian/mechanistic target of rapamycin (mTOR), phosphorylated mTOR (p-mTOR), S6, p-S6, phosphatase and tensin homolog (PTEN), p-PTEN, B-Raf, p-B-Raf, p38 mitogen-activated protein kinase (MAPK), p-p38 MAPK, p44/42, p-p44/42 and β-actin (Cell Signaling Technology, Danvers, MA, USA). Membranes were washed in Tris-buffered saline containing 0.1% Tween-20 (TBS-T) buffer and then incubated for 1 h at room temperature with the appropriate horseradish peroxidase (HRP)-conjugated secondary antibodies (1:2000 dilution; Bio-Rad Laboratories, Irvine, CA, USA). After final washes in TBS-T, immunoreactive proteins were detected using enhanced chemiluminescence (ECL) reagents (Thermo Scientific) followed by exposure to X-ray film (Amersham Pharmacia Biotech Inc., Buckinghamshire, UK).

### 4.7. Statistical Analyses

PDX engraftment and clinical features of patients were compared using the Wilcoxon rank-sum test, Pearson chi-squared test and Fisher’s exact test. Overall survival and disease-free survival according to breast cancer PDX engraftment status were assessed using Kaplan–Meier plots and the log-rank test. Overall survival was measured from the date of diagnosis to date of death or last follow-up. Disease-free survival was calculated from the date of diagnosis to the day of recurrence. A *p*-value ≤0.05 was considered statistically significant. GraphPad Prism (Version 5.03, GraphPad Software, San Diego, CA, USA) was used for all statistical analyses.

## 5. Conclusions

The PDX model offers considerable advantages in preclinical studies of new therapeutic agents or for screening and testing appropriate cancer drug for individual patients, reflecting the fact that histopathological features of patient tumors are retained after passage in mice [23]. We found that it was possible to select treatment targets using an analysis of gene expression panels and test potential treatment drugs using PDX models. Further technical improvements that expedite PDX generation and a more rapid system for evaluating treatment effects on PDX models are required for effective clinical application. It will also prove necessary to develop methods for stable storage and successful transplantation of patients’ tumor tissues to efficiently produce PDX models of individual patients.

## Figures and Tables

**Table 1:** Clinical characteristics of patients according to engraftment status.

Characteristics	Total (%)	Engrafted (%)	Failed (%)	*p*-Value
Number of patients	114 (100)	20 (17.5)	94 (82.5)	
Age at collection (years)	49.60 ± 11.60	46.40 ± 13.69	49.94 ± 10.90	0.175 ^(b)^
Tissue source				
Surgery	57 (50.0)	14 (24.6)	43 (75.4)	0.085 ^(c)^
Biopsy	57 (50.0)	6 (10.5)	51 (89.5)	
Stage				
I	9 (7.9)	1 (11.1)	8 (88.9)	0.039 ^(d)^
II	47 (41.2)	5 (10.6)	42(89.4)	
III	48 (42.1)	9 (18.7)	39(81.3)	
IV	10 (8.8)	5 (50.0)	5 (50.0)	
Chemotherapy resistance ^(a)^				
Yes	28 (24.6)	13 (46.4)	15 (53.6)	<0.001 ^(d)^
No	86 (74.6)	7 (8.1)	79 (91.9)	

Values are presented as numbers (%) or means ± standard deviation. ^(a)^ Chemotherapy resistance is defined as patients with progression on chemotherapy or recurrence within one year after adjuvant chemotherapy. ^(b)^ Wilcoxon rank-sum test. ^(c)^ Pearson chi-square test. ^(d)^ Fisher’s exact test.

## Supplementary Materials

The following are available online at https://www.mdpi.com/2072-6694/11/4/574/s1: Figure S1: Changes in body weight in PDX model mice following treatment with drugs; Figure S2: In vivo efficacy of sorafenib and everolimus against PDX models from PT12 (TNBC subtype); Figure S3: Gene expression analysis using a Nanostring nCounter GX human cancer reference kit and efficacy tests of PX-478 and neratinib against PDX models from PT5 (HR^−^/HER2^+^ subtype); Table S1: Clinical features of patient-derived xenograft tumors from 17 patients.

## References

[1] May M. (2018). Cancer research with a human touch. Nature.

[2] Barretina J., Caponigro G., Stransky N., Venkatesan K., Margolin A.A., Kim S., Wilson C.J., Lehar J., Kryukov G.V., Sonkin D. (2012). The Cancer Cell Line Encyclopedia enables predictive modelling of anticancer drug sensitivity. Nature.

[3] Jung J., Seol H.S., Chang S. (2018). The Generation and Application of Patient-Derived Xenograft Model for Cancer Research. Cancer Res. Treat..

[4] Whittle J.R., Lewis M.T., Lindeman G.J., Visvader J.E. (2015). Patient-derived xenograft models of breast cancer and their predictive power. Breast Cancer Res..

[5] Zhang X., Claerhout S., Prat A., Dobrolecki L.E., Petrovic I., Lai Q., Landis M.D., Wiechmann L., Schiff R., Giuliano M. (2013). A renewable tissue resource of phenotypically stable, biologically and ethnically diverse, patient-derived human breast cancer xenograft models. Cancer Res..

[6] Eyre R., Alferez D.G., Spence K., Kamal M., Shaw F.L., Simoes B.M., Santiago-Gomez A., Sarmiento-Castro A., Bramley M., Absar M. (2016). Patient-derived Mammosphere and Xenograft Tumour Initiation Correlates with Progression to Metastasis. J. Mammary Gland Biol. Neoplasia.

[7] Li S., Shen D., Shao J., Crowder R., Liu W., Prat A., He X., Liu S., Hoog J., Lu C. (2013). Endocrine-therapy-resistant ESR1 variants revealed by genomic characterization of breast-cancer-derived xenografts. Cell Rep..

[8] Kabos P., Finlay-Schultz J., Li C., Kline E., Finlayson C., Wisell J., Manuel C.A., Edgerton S.M., Harrell J.C., Elias A. (2012). Patient-derived luminal breast cancer xenografts retain hormone receptor heterogeneity and help define unique estrogen-dependent gene signatures. Breast Cancer Res. Treat.

[9] Zhang H., Cohen A.L., Krishnakumar S., Wapnir I.L., Veeriah S., Deng G., Coram M.A., Piskun C.M., Longacre T.A., Herrler M. (2014). Patient-derived xenografts of triple-negative breast cancer reproduce molecular features of patient tumors and respond to mTOR inhibition. Breast Cancer Res..

[10] DeRose Y.S., Wang G., Lin Y.C., Bernard P.S., Buys S.S., Ebbert M.T., Factor R., Matsen C., Milash B.A., Nelson E. (2011). Tumor grafts derived from women with breast cancer authentically reflect tumor pathology, growth, metastasis and disease outcomes. Nat. Med..

[11] Reyal F., Guyader C., Decraene C., Lucchesi C., Auger N., Assayag F., De Plater L., Gentien D., Poupon M.F., Cottu P. (2012). Molecular profiling of patient-derived breast cancer xenografts. Breast Cancer Res..

[12] Dzobo K., Rowe A., Senthebane D.A., AlMazyadi M.A.M., Patten V., Parker M.I. (2018). Three-Dimensional Organoids in Cancer Research: The Search for the Holy Grail of Preclinical Cancer Modeling. OMICS.

[13] Drost J., Clevers H. (2018). Organoids in cancer research. Nat. Rev. Cancer.

[14] Niu G., Chen X. (2010). Vascular endothelial growth factor as an anti-angiogenic target for cancer therapy. Curr. Drug Targets.

[15] Shibuya M., Claesson-Welsh L. (2006). Signal transduction by VEGF receptors in regulation of angiogenesis and lymphangiogenesis. Exp. Cell Res..

[16] Montero A.J., Escobar M., Lopes G., Gluck S., Vogel C. (2012). Bevacizumab in the treatment of metastatic breast cancer: Friend or foe?. Curr. Oncol. Rep..

[17] Tiainen L., Tanner M., Lahdenpera O., Vihinen P., Jukkola A., Karihtala P., Paunu N., Huttunen T., Kellokumpu-Lehtinen P.L. (2016). Bevacizumab Combined with Docetaxel or Paclitaxel as First-line Treatment of HER2-negative Metastatic Breast Cancer. Anticancer Res..

[18] Pories S.E., Wulf G.M. (2010). Evidence for the role of bevacizumab in the treatment of advanced metastatic breast cancer: A review. Breast Cancer (Dove Med. Press).

[19] Koh M.Y., Spivak-Kroizman T., Venturini S., Welsh S., Williams R.R., Kirkpatrick D.L., Powis G. (2008). Molecular mechanisms for the activity of PX-478, an antitumor inhibitor of the hypoxia-inducible factor-1alpha. Mol. Cancer Ther..

[20] Macpherson G.R., Figg W.D. (2004). Small molecule-mediated anti-cancer therapy via hypoxia-inducible factor-1 blockade. Cancer Biol. Ther..

[21] Welsh S., Williams R., Kirkpatrick L., Paine-Murrieta G., Powis G. (2004). Antitumor activity and pharmacodynamic properties of PX-478, an inhibitor of hypoxia-inducible factor-1alpha. Mol. Cancer Ther..

[22] Choi S.Y., Lin D., Gout P.W., Collins C.C., Xu Y., Wang Y. (2014). Lessons from patient-derived xenografts for better in vitro modeling of human cancer. Adv. Drug Deliv. Rev..

[23] Kawaguchi T., Foster B.A., Young J., Takabe K. (2017). Current Update of Patient-Derived Xenograft Model for Translational Breast Cancer Research. J. Mammary Gland Biol. Neoplasia.

[24] Marangoni E., Vincent-Salomon A., Auger N., Degeorges A., Assayag F., de Cremoux P., de Plater L., Guyader C., De Pinieux G., Judde J.G. (2007). A new model of patient tumor-derived breast cancer xenografts for preclinical assays. Clin. Cancer Res..

[25] Moon H.G., Oh K., Lee J., Lee M., Kim J.Y., Yoo T.K., Seo M.W., Park A.K., Ryu H.S., Jung E.J. (2015). Prognostic and functional importance of the engraftment-associated genes in the patient-derived xenograft models of triple-negative breast cancers. Breast Cancer Res. Treat.

[26] Yu J., Qin B., Moyer A.M., Sinnwell J.P., Thompson K.J., Copland J.A., Marlow L.A., Miller J.L., Yin P., Gao B. (2017). Establishing and characterizing patient-derived xenografts using pre-chemotherapy percutaneous biopsy and post-chemotherapy surgical samples from a prospective neoadjuvant breast cancer study. Breast Cancer Res..

[27] Kuperwasser C., Chavarria T., Wu M., Magrane G., Gray J.W., Carey L., Richardson A., Weinberg R.A. (2004). Reconstruction of functionally normal and malignant human breast tissues in mice. Proc. Natl. Acad. Sci. USA.

[28] DeRose Y.S., Gligorich K.M., Wang G., Georgelas A., Bowman P., Courdy S.J., Welm A.L., Welm B.E. (2013). Patient-derived models of human breast cancer: Protocols for in vitro and in vivo applications in tumor biology and translational medicine. Curr. Protoc. Pharm..

[29] Quintana E., Shackleton M., Sabel M.S., Fullen D.R., Johnson T.M., Morrison S.J. (2008). Efficient tumour formation by single human melanoma cells. Nature.

[30] Wilhelm S.M., Adnane L., Newell P., Villanueva A., Llovet J.M., Lynch M. (2008). Preclinical overview of sorafenib, a multikinase inhibitor that targets both Raf and VEGF and PDGF receptor tyrosine kinase signaling. Mol. Cancer Ther..

[31] Wilhelm S.M., Carter C., Tang L., Wilkie D., McNabola A., Rong H., Chen C., Zhang X., Vincent P., McHugh M. (2004). BAY 43-9006 exhibits broad spectrum oral antitumor activity and targets the RAF/MEK/ERK pathway and receptor tyrosine kinases involved in tumor progression and angiogenesis. Cancer Res..

[32] Porta C., Paglino C., Mosca A. (2014). Targeting PI3K/Akt/mTOR Signaling in Cancer. Front. Oncol..

[33] Masoud G.N., Li W. (2015). HIF-1alpha pathway: Role, regulation and intervention for cancer therapy. Acta Pharm. Sin. B.

[34] Houghton P.J. (2010). Everolimus. Clin. Cancer Res..

[35] Miller K., Wang M., Gralow J., Dickler M., Cobleigh M., Perez E.A., Shenkier T., Cella D., Davidson N.E. (2007). Paclitaxel plus bevacizumab versus paclitaxel alone for metastatic breast cancer. N. Engl. J. Med..

[36] Whelan K.A., Schwab L.P., Karakashev S.V., Franchetti L., Johannes G.J., Seagroves T.N., Reginato M.J. (2013). The oncogene HER2/neu (ERBB2) requires the hypoxia-inducible factor HIF-1 for mammary tumor growth and anoikis resistance. J. Biol. Chem..

[37] Chen J., Imanaka N., Chen J., Griffin J.D. (2010). Hypoxia potentiates Notch signaling in breast cancer leading to decreased E-cadherin expression and increased cell migration and invasion. Br. J. Cancer.

[38] West M., Blanchette C., Dressman H., Huang E., Ishida S., Spang R., Zuzan H., Olson J.A., Marks J.R., Nevins J.R. (2001). Predicting the clinical status of human breast cancer by using gene expression profiles. Proc. Natl. Acad. Sci. USA.

[39] Jwa E., Shin K.H., Kim J.Y., Park Y.H., Jung S.Y., Lee E.S., Park I.H., Lee K.S., Ro J., Kim Y.J. (2016). Locoregional Recurrence by Tumor Biology in Breast Cancer Patients after Preoperative Chemotherapy and Breast Conservation Treatment. Cancer Res. Treat.

[40] Lee M.H., Jung S.Y., Kang S.H., Song E.J., Park I.H., Kong S.Y., Kwon Y.M., Lee K.S., Kang H.S., Lee E.S. (2016). The Significance of Serum HER2 Levels at Diagnosis on Intrinsic Subtype-Specific Outcome of Operable Breast Cancer Patients. PLoS ONE.

[41] Hammond M.E., Hayes D.F., Dowsett M., Allred D.C., Hagerty K.L., Badve S., Fitzgibbons P.L., Francis G., Goldstein N.S., Hayes M. (2010). American Society of Clinical Oncology/College Of American Pathologists guideline recommendations for immunohistochemical testing of estrogen and progesterone receptors in breast cancer. J. Clin. Oncol..

[42] Wolff A.C., Hammond M.E., Hicks D.G., Dowsett M., McShane L.M., Allison K.H., Allred D.C., Bartlett J.M., Bilous M., Fitzgibbons P. (2013). Recommendations for human epidermal growth factor receptor 2 testing in breast cancer: American Society of Clinical Oncology/College of American Pathologists clinical practice guideline update. J. Clin. Oncol..

