# Supplementary material for: Integrative In Vivo Drug Testing Using Gene Expression Signature and Patient-Derived Xenografts from Treatment-Refractory HER2 Positive and Triple-Negative Subtypes of Breast Cancer
Source: Cancers (Basel). 2019 Apr 23;11(4):574. doi: 10.3390/cancers11040574 (PMC6520730; doi:10.3390/cancers11040574)
Supplement: Supplementary file 1 [file cancers-11-00574-s001.pdf]

Supplementary Materials: Integrative In Vivo Drug Testing Using Gene Expression Signature and Patient-Derived Xenografts from Treatment-Refractory HER2 Positive and Triple-Negative Subtypes of Breast Cancer

Jin-Sun Ryu, Sung Hoon Sim, In Hae Park, Eun Gyeong Lee, Eun Sook Lee, Yun-Hee Kim, Youngmee Kwon, Sun-Young Kong and Keun Seok Lee

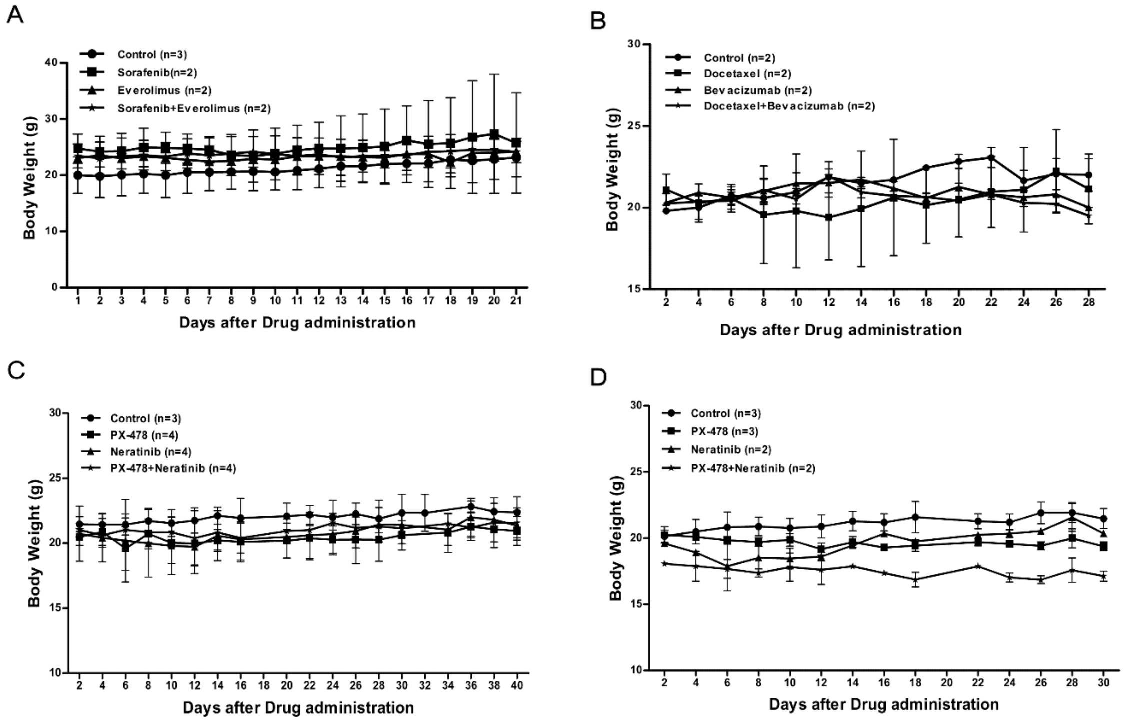

Figure S1. Changes in body weight in PDX model mice following treatment with drugs. (**A**) Body weights of patient-derived xenograft (PDX) model mice established from PT14, monitored every day, are presented as means ± SD. Female mice were treated with sorafenib (●), everolimus (▲), or their combination (★); mice administered saline (■) served as controls. (**B**) Body weights of PDX model mice established from PT12, monitored three times a week, are presented as means ± SD. Female mice were treated with docetaxel (●, 3 mg/kg), bevacizumab (▲, 5 mg/kg), or their combination (★) by intraperitoneal injection three times a week for 4 weeks; mice administered saline (■) served as controls. (**C**, **D**) Weights of PDX tumors from PT9 and PT10, monitored three times a week, are presented as means ± SD. Female mice were administered PX-478 (●, 10 mg/kg), neratinib (▲, 20 mg/kg), or their combination (★), via oral gavage three times a week in female mice for 40 days (C) and 30 days (D); mice administered saline (■) served as controls.

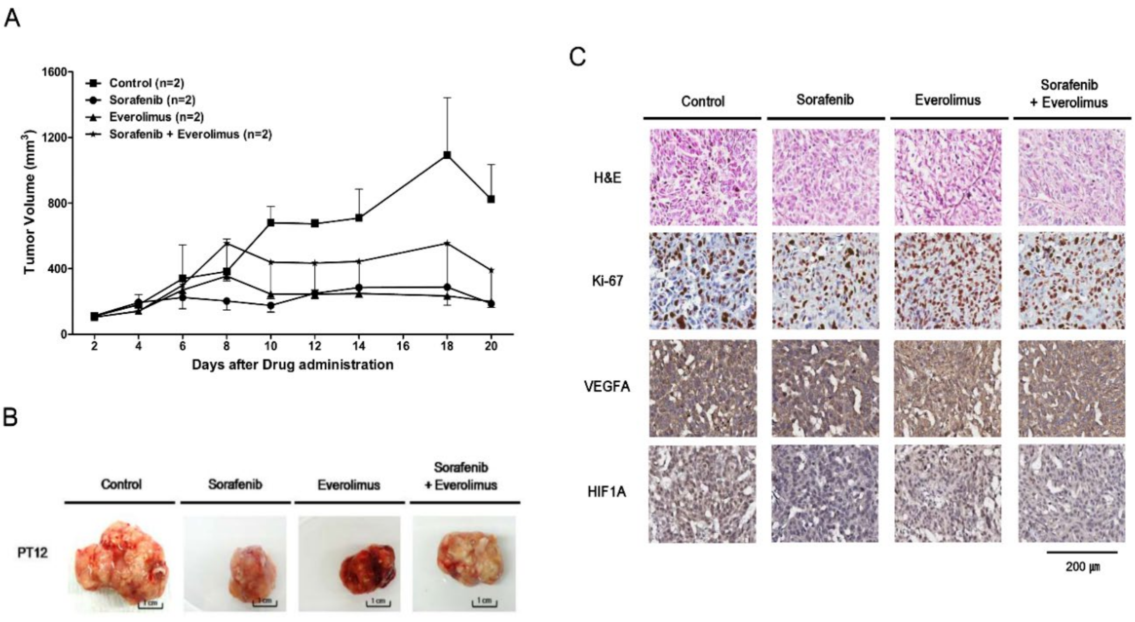

Figure S2. In vivo efficacy of sorafenib and everolimus against PDX models from PT12 (TNBC subtype) (**A**) Tumor volumes (F2) were determined in female mice (n = 2) treated with sorafenib (●, 120 mg/kg), everolimus (▲, 20 mg/kg), or their combination (★), given orally by gavage once a day; mice administered saline (■) served as controls. Tumor volumes are presented as means ± SD; p-values (unpaired t-test) at 20 days are shown ($p > 0.2$ for control vs. sorafenib, control vs. everolimus and control vs. sorafenib + everolimus). (**B**) Images of PT12 PDX model tumors from mice treated with sorafenib, everolimus, or their combination. (**C**) H&E staining and immunohistochemical analyses of Ki-67, VEGFA, and HIF1A in PT12 PDX tumors after treatment of tumor-bearing mice with drugs. Scale bar, 200 μm.

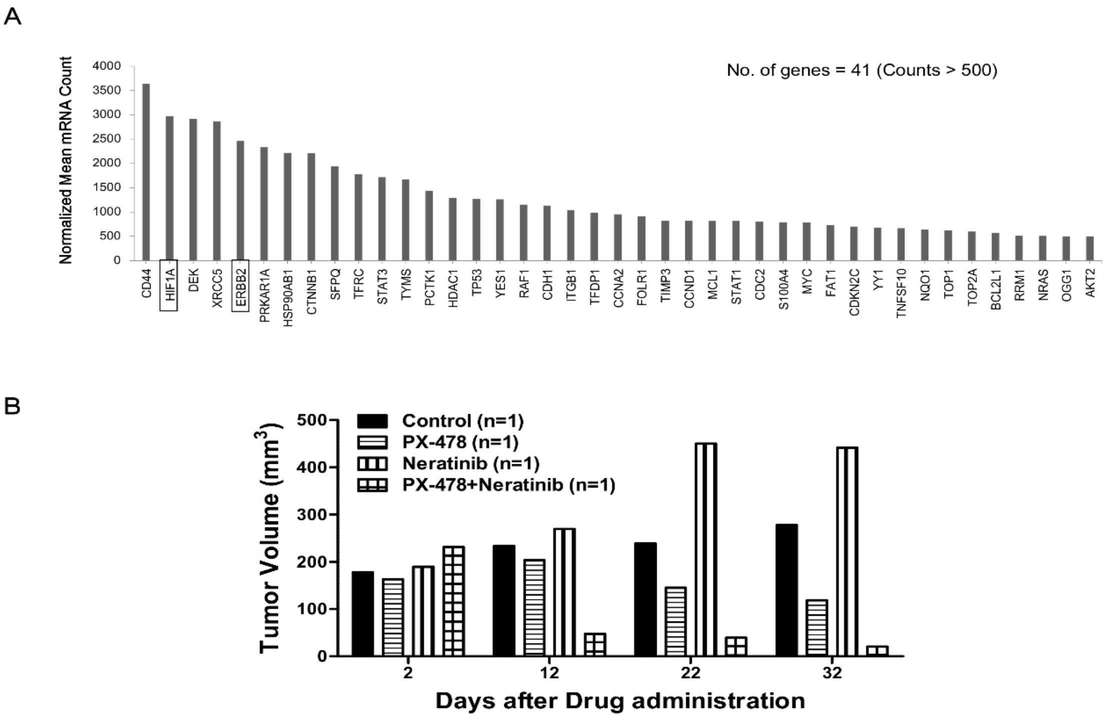

Figure S3. Gene expression analysis using a Nanostring nCounter GX human cancer reference kit and efficacy tests of PX-478 and neratinib against PDX models from PT5 (HR^{-}/HER2^{+} subtype). (**A**) The Nanostring nCounter System was used to examine gene expression profiles of PDX tumors (F2) from

PT5. The top upregulated 41 genes with counts >500 were selected among the 230 human cancer-related genes. (**B**) Tumor volumes (F4) were measured at 2, 12, 22, and 32 days in female mice ($n = 1$) treated with PX-478 (▤, 30 mg/kg), neratinib (▥, 40 mg/kg), or their combination (▦), given orally by gavage, every other day; mice administered saline (■) served as controls.

Table S1. Clinical features of patient-derived xenograft tumors from 17 patients.

No.	Patient	ER	PR	HER2	Tumor site/Source	Histology	Days to generate 100 mm^3 F1 tumors (p = 0.830 *)
1	PT1	+	+	−	Breast (Rt.)/surgery	IDC	37
2	PT2	+	+	+	LN (Lt. axilla)/biopsy	IDC	23
3	PT3	+	−	+	Breast (Rt.)/surgery	IDC	26
4	PT4	+	+	+	Chest wall (Lt.)/biopsy	IDC	186
5	PT5	−	−	+	Breast (Rt.)/biopsy	IDC	94
6	PT5	−	−	+	Breast (Rt.)/surgery	IDC	55
7	PT6	−	−	+	Breast (Lt.)/biopsy	IDC	33
8	PT7	−	−	+	Breast (Lt.)/surgery	IDC	68
9	PT8	−	−	+	Breast (Rt.)/surgery	IDC	5
10	PT9	−	−	+	Breast (Lt.)/surgery	IDC	71
11	PT10	−	−	+	Breast (Rt.)/surgery	Mucinous	137
12	PT11	−	−	−	LN (Lt. axilla)/biopsy	IDC	112
13	PT11	−	−	−	Breast (Lt.)/surgery	IDC	55
14	PT12	−	−	−	Breast (Rt.)/biopsy	IDC	51
15	PT12	−	−	−	Breast (Rt.)/surgery	IDC	78
16	PT13	−	−	−	Breast (Rt.)/surgery	IDC	64
17	PT14	−	−	−	Breast (Lt.)/surgery	IDC	35
18	PT15	−	−	−	LN (Neck)/surgery	IDC	52
19	PT16	−	−	−	Breast (Rt.)/surgery	IDC	20
20	PT17	−	−	−	Breast (Rt.)/surgery	IDC	47

ER, estrogen receptor; PR, progesterone receptor; HER2, human epidermal growth factor 2; IDC, invasive ductal carcinoma; Rt., right; Lt., left. * Kruskal–Wallis test.

© 2019 by the authors. Licensee MDPI, Basel, Switzerland. This article is an open access article distributed under the terms and conditions of the Creative Commons Attribution (CC BY) license (http://creativecommons.org/licenses/by/4.0/).
